# Supplementary material for: Multi‐dataset identification of innovative feature genes and molecular mechanisms in keratoconus
Source: J Cell Mol Med. 2024 Sep 19;28(18):e70079. doi: 10.1111/jcmm.70079 (PMC11412914; doi:10.1111/jcmm.70079)
Supplement: Supplementary file 2 — Material S2. [file JCMM-28-e70079-s002.zip › 7.GSEA analysis/1.ARL11/KEGG_RENIN_ANGIOTENSIN_SYSTEM.html]

Details for gene set KEGG\_RENIN\_ANGIOTENSIN\_SYSTEM[GSEA]

|  || Dataset | ARL11.group.cls#h\_versus\_l |
| Phenotype | group.cls#h\_versus\_l |
| Upregulated in class | l |
| GeneSet | KEGG\_RENIN\_ANGIOTENSIN\_SYSTEM |
| Enrichment Score (ES) | -0.64088184 |
| Normalized Enrichment Score (NES) | -1.4981483 |
| Nominal p-value | 0.05788423 |
| FDR q-value | 1.0 |
| FWER p-Value | 0.774 |
Table: GSEA Results Summary

  

Fig 1: Enrichment plot: KEGG\_RENIN\_ANGIOTENSIN\_SYSTEM      
 Profile of the Running ES Score & Positions of GeneSet Members on the Rank Ordered List

  

| SYMBOL | TITLE | RANK IN GENE LIST | RANK METRIC SCORE | RUNNING ES | CORE ENRICHMENT || 1 | AGTR2 | na | 11576 | 0.041 | -0.3136 | No |
| 2 | LNPEP | na | 12369 | 0.033 | -0.3205 | No |
| 3 | ACE | na | 14186 | 0.017 | -0.3643 | No |
| 4 | ACE2 | na | 14988 | 0.011 | -0.3819 | No |
| 5 | THOP1 | na | 16318 | 0.002 | -0.4194 | No |
| 6 | CTSA | na | 16614 | -0.001 | -0.4276 | No |
| 7 | MAS1 | na | 20889 | -0.030 | -0.5359 | No |
| 8 | CPA3 | na | 22051 | -0.040 | -0.5495 | No |
| 9 | CTSG | na | 23366 | -0.054 | -0.5609 | No |
| 10 | ANPEP | na | 26145 | -0.093 | -0.5955 | Yes |
| 11 | NLN | na | 27653 | -0.122 | -0.5791 | Yes |
| 12 | ENPEP | na | 28424 | -0.142 | -0.5320 | Yes |
| 13 | MME | na | 29457 | -0.171 | -0.4781 | Yes |
| 14 | AGTR1 | na | 32018 | -0.261 | -0.4242 | Yes |
| 15 | REN | na | 32393 | -0.280 | -0.2978 | Yes |
| 16 | AGT | na | 33083 | -0.328 | -0.1572 | Yes |
| 17 | CMA1 | na | 33985 | -0.418 | 0.0214 | Yes |
Table: GSEA details [plain text format]

  

Fig 2: KEGG\_RENIN\_ANGIOTENSIN\_SYSTEM      
 Blue-Pink O' Gram in the Space of the Analyzed GeneSet

  

Fig 3: KEGG\_RENIN\_ANGIOTENSIN\_SYSTEM: Random ES distribution      
 Gene set null distribution of ES for **KEGG\_RENIN\_ANGIOTENSIN\_SYSTEM**
